# Safety assessment of disulfiram: real-world adverse event analysis based on FAERS database

**DOI:** 10.3389/fpsyt.2024.1498204

**Published:** 2024-11-19

**Authors:** Jing Luo, Yaqi Zeng, Zhe Chen, Yaan Luo, Li Shi, Xuhui Zhou

**Affiliations:** ^1^ Department of Psychiatry, The School of Clinical Medicine, Hunan University of Chinese Medicine, Changsha, Hunan, China; ^2^ Department of Psychiatry, The Second People's Hospital of Hunan Province (Brain Hospital of Hunan Province), Changsha, Hunan, China; ^3^ Department of Thoracic Surgery, The Second Xiangya Hospital, Central South University, Changsha, Hunan, China

**Keywords:** disulfiram, FAERS, real-world data analysis, signal mining, adverse event

## Abstract

**Objective:**

Disulfiram, an FDA-approved medication for AUD, has shown significant potential as a repurposed drug in therapeutic areas including oncology and infectious diseases. The purpose of study is to analyze adverse events (AEs) associated with disulfiram by examining the FAERS database, with a focus on understanding its safety profile in both traditional and emerging applications.

**Methods:**

AE reports concerning disulfiram in the FAERS database from the fourth quarter of 2002 to the third quarter of 2023 were extracted. Various signal detection methods, including ROR, PRR, BCPNN, and MGPS, were used to detect and categorize adverse events.

**Results:**

The study collected 52,159,321 AE reports, with 508 reports primarily suspecting disulfiram, identifying 104 Preferred Terms (PTs) across 25 System Organ Classes (SOCs). Major categories of AEs included off label use, psychiatric symptom, liver transplant, and polyneuropathy, with off label use being notably the most reported issue. Strong and new potential AEs were identified, including neurological and psychiatric issues like hypomania, delirium, and vocal cord paralysis; cardiac issues such as electrocardiogram st segment depression; and off label use-related issues like Jarisch-Herxheimer reaction.

**Conclusion:**

Disulfiram poses risks of various adverse reactions while having promise as a “repurposed” agent. In clinical applications, practitioners should closely monitor occurrences of hepatobiliary disorders, psychiatric disorders, and nervous system disorders.

## 1 Introduction

Disulfiram is an electrophilic quaternary ammonium compound whose metabolite diethyldithiocarbamate (DDTC) binds to metal ions (e.g., copper) and forms a precipitate. DDTC can hinder the activity of different metabolic enzymes in the body, such as aldehyde dehydrogenase (ALDH), by attaching to important cofactors (1). When alcohol is consumed, the suppression of ALDH causes acetaldehyde to accumulate, resulting in unpleasant symptoms like nausea, vomiting, and headaches (2). A significant degree of pain may be caused by even a small amount of alcohol, which is one of the reasons why individuals quit drinking alcohol. As a pharmacological therapy for alcohol use disorder (AUD), disulfiram was approved by the Food and Drug Administration (FDA) of the United States of America in the year 1951. As a result of the detrimental effects of disulfiram, which include hepatotoxicity and neurotoxicity, its application in clinical settings has been reduced. Due to the fact that these toxicities have the potential to result in significant health consequences, the employment of this drug in alcohol cessation therapy is rather uncommon. Since pharmaceutical treatments for AUD, such as naltrexone and acamprosate, have received approval from the FDA, there has been a decrease in the utilization of disulfiram (3).

Due to the growing interest in pharmacological repurposing, researchers have re-evaluated the therapeutic potential of disulfiram. Disulfiram possesses sulfhydryl-modifying and chelating properties that causes a diverse range of pharmacological effects, such as anticancer, antiparasitic, and anti-inflammatory applications (4–6). Multiple clinical trials have been registered for new indications, presenting possibilities for disulfiram’s use in new therapeutic areas (7, 8). For instance, a phase I/II clinical trial was conducted to evaluate the safety and preliminary efficacy of disulfiram combined with copper in patients with newly diagnosed glioblastoma. The findings indicated that promising responses were observed in patients with BRAF-mutant GBM (9). Another study demonstrated that disulfiram combined with copper ions forms the Cu(DDC)2 complex. Cu(DDC)2 induces apoptosis in tumor cells and promotes the production of reactive oxygen species (ROS), showing antitumor effects even in drug-resistant cancer cells (10). Nevertheless, repurposing disulfiram proves challenging due to the drug’s plethora of undesirable side effects. The combination of alcohol with various everyday household products, such as cough syrups and hand sanitizers containing alcohol, can result in a disulfiram-alcohol-like reaction. As a result, employing it in the clinic is more complicated. Before considering its application to new indications, a comprehensive risk assessment is essential (11).

Recently, a pharmacovigilance study analyzed adverse drug reaction data from the International Psychiatric Medication Safety Program (AMSP). According to Greil et al., an increased risk of cutaneous adverse drug reactions (CADR) is associated with disulfiram (12). But additional systemic side effects of disulfiram were not included in this research. This calls for more investigation. The FDA Adverse Event Reporting System (FAERS) is a database that collects information on adverse events (AEs) and medication errors associated with drugs and biologic products. It helps the FDA monitor and review the safety of these products and identify potential safety issues. This study was based on the FAERS database and used multiple signaling methods to detect the AEs signals of disulfiram. The primary objective of this research is to shed light on its safety profile in both traditional and emerging applications.

## 2 Materials and methods

### 2.1 Data sources and processing

In the FAERS database, reporters can explicitly indicate the role of a drug in the occurrence of an adverse event, such as “Primary Suspect” (PS), “Secondary Suspect” (SS), “Interaction,” or “Concomitant” (C). This study gathered all AE reports from the fourth quarter of 2002 to the third quarter of 2023 in which disulfiram was the primary suspected drug. All data were sourced from the FAERS database. The study collected clinical features of patients who experienced AEs related to disulfiram, including gender, age, reporter type, country of report, year of report, adverse events and their outcomes. To eliminate duplicate reports, we performed data cleaning following the FDA’s recommended approach. Firstly, the Demo table was sorted by CASEID, FDA_DT, and PRIMARYID. For records with the same CASEID, we retained the most recent report based on the update date. Secondly, when FDA_DT and CASEID were identical, we selected the report with the largest PRIMARYID value. The analysis utilized the Medical Dictionary for Regulatory Activities (MedDRA) for coding, categorizing, and localizing AEs signals according to Preferred Terms (PTs) and System Organ Classes (SOCs) categories (13). PTs with reported counts of ≥3 were included.

### 2.2 Statistical analysis

All AE reports associated with disulfiram were analyzed using descriptive statistics. Multiple disproportionality methods were utilized to detect drug-AE signals, including Reporting Odds Ratio (ROR), Proportional Reporting Ratio (PRR), Bayesian Confidence Propagation Neural Network (BCPNN), and Multi-Item Gamma Poisson Shrinker (MGPS) (14–17). ROR and PRR highlighted risks associated with specific drugs by identifying abnormally high proportions of AE reports. ROR corrected biases from small sample sizes, while PRR provided greater specificity in distinguishing drug-related AEs. BCPNN integrated multi-source data and cross-validation, offering more credible drug-AE associations. MGPS accounted for report quantity and background risk, detecting rare event signals. By combining these methods, the study expanded detection scope, verified results, and leveraged each method’s strengths to identify more complete and trustworthy safety signals. The combined use of these algorithms enables cross-validation to reduce erroneous signals or irrelevant reports. Moreover, by adjusting thresholds and variance within the algorithms, rare but significant adverse event signals can be more effectively identified. This approach enhances the sensitivity and accuracy in detecting these rare events. Detailed calculation formulas and specific procedures are presented in **Tables 1**, **2**. We used Microsoft Excel 2021 and R software 4.3.1 to conduct our statistical analyses.

**Table 1:** Four grid table.

	Target AEs	Non-target AEs	Total
disulfiram	a	b	a+b
Non- disulfiram	c	d	c+d
Total	a+c	b+d	N = a + b + c + d

AE, adverse event.

N, the number of reports; a is the number of cases where a specific adverse event occurred after using disulfiram, b is the number of cases where disulfiram was used but the specific adverse event did not occur, c is the number of cases where the specific adverse event occurred without the use of disulfiram, d is the number of cases where neither disulfiram was used nor the specific adverse event occurred.

**Table 2:** ROR, PRR, BCPNN, and MGPS methods, formulas and thresholds.

Method	Formula	Threshold
ROR	$\text{ROR}=\frac{\left(a/c\right)}{\left(b/d\right)}=\frac{ad}{bc}$ $\text{SE}\left(\text{lnROR}\right)=\sqrt{\left(\frac{1}{a}+\frac{1}{b}+\frac{1}{c}+\frac{1}{d}\right)}$ $95\text{\%CI}=e^{\ln\left(ROR\right)\pm 1.96\sqrt{\left(\frac{1}{a}+\frac{1}{b}+\frac{1}{c}+\frac{1}{d}\right)}}$	a ≥ 3 and 95 % CI (lower limit) >1
PRR	$\text{PRR}=\frac{a/\left(a+b\right)}{c/\left(c+d\right)}$ $\text{SE}(\ln PRR)=\sqrt{\left(\frac{1}{a}-\frac{1}{a+b}+\frac{1}{c}-\frac{1}{c+d}\right)}$ $95\text{\%CI}=e^{\ln\left(PRR\right)\pm 1.96\sqrt{\left(\frac{1}{a}-\frac{1}{a+b}+\frac{1}{c}-\frac{1}{c+d}\right)}}$	a≥3, PRR≥2, ${\text{x}}^{2}$ ≥4
BCPNN	$\text{IC}=\log_{2}\frac{p\left(x,y\right)}{p\left(x\right)p\left(y\right)}=\log_{2}\frac{a\left(a+b+c+d\right)}{\left(a+b\right)\left(a+c\right)}$ $\text{E}\left(\text{IC}\right)=\log_{2}\frac{\left(a+\gamma ij\right)\left(a+b+c+d+\alpha \right)\left(a+b+c+d+\beta \right)}{\left(a+b+c+d+\gamma \right)\left(a+b+\alpha i\right)\left(a+c+\beta j\right)}$ $\text{V}\left(\text{IC}\right)=\frac{1}{{\left(\ln2\right)}^{2}}\left\{\left[\frac{\left(a+b+c+d\right)-a+\gamma -\gamma ij}{\left(a+\gamma ij\right)\left(1+a+b+c+d+\gamma \right)}\right]+\left[\frac{\left(a+b+c+d\right)-\left(a+b\right)+\alpha -\alpha i}{\left(a+b+\alpha i\right)\left(1+a+b+c+d+\alpha \right)}\right]+\left[\frac{\left(a+b+c+d\right)-\left(a+c\right)+\beta -\beta j}{\left(a+c+\beta j\right)\left(1+a+b+c+d+\beta \right)}\right]\right\}$ $\gamma =\gamma ij\frac{\left(a+b+c+d+\alpha \right)+\left(a+b+c+d+\beta \right)}{\left(a+b+\alpha i\right)\left(a+c+\beta j\right)}$ $\text{IC}-2\text{SD}=\text{E}\left(\text{IC}\right)-2\sqrt{V\left(IC\right)}$	IC025 > 0
MGPS	$\text{EBGM}=\frac{a\left(a+b+c+d\right)}{\left(a+c\right)\left(a+b\right)}$ $95\text{\%CI}=e^{\ln\left(EBGM\right)\pm 1.96\sqrt{\left(\frac{1}{a}+\frac{1}{b}+\frac{1}{c}+\frac{1}{d}\right)}}$	EBGM05 > 2

BCPNN, Bayesian confidence propagation neural network; CI, confidence interval; EBGM, empirical Bayesian geometric mean; EEBGM05, the lower limit of the 95% CI, for EBGM; IC, information component; IC025, the lower limit of 95% CI, for the IC; E(IC), the IC, expectations; V(IC), the variance of IC; MGPS, Multi-Item Gamma Poisson Shrinker; PRR, proportional reporting ratio; ROR, reporting odds ratio; SD, standard deviation; SE(InROR), standard error of the InROR; γ, γij represent the parameters of the Dirichlet distribution; α, αi, β, βj represent the parameters of the Beta distribution;χ2, chi-squared.

a is the number of cases where a specific adverse event occurred after using disulfiram, b is the number of cases where disulfiram was used but the specific adverse event did not occur, c is the number of cases where the specific adverse event occurred without the use of disulfiram, d is the number of cases where neither disulfiram was used nor the specific adverse event occurred.

## 3 Results

### 3.1 Basic characteristic of AE reports

This study collected a total of 52,159,321 AE reports from the fourth quarter of 2002 to the third quarter of 2023 in the FAERS database. There were 508 reports primarily suspecting disulfiram among these. Based on the gender of reports, male reporters (298, accounting for 58.7%) significantly outnumbered female reporters (173, accounting for 34.1%). Age-wise, the majority of reporters belonged to the 18-64 age range, accounting for 72.6%. The main sources of these reports were Physician, who made up 27%. Additionally, patients themselves and health professionals each contributed approximately 20% of the reports. Regarding the reporting countries, the foremost countries contributing to AE records of disulfiram were the United States, followed by Sweden, India and the United Kingdom, accounting for 226, 40, 34, and 34 records, respectively. From 2014 to 2020, the number of disulfiram adverse event reports generally declined, while in 2021, there was a significant increase. From the perspective of adverse outcomes, hospitalization or prolongation of hospitalization accounted for 36.5% of all reports. Furthermore, other serious accounted for 38.6%. This information is summarized in **Table 3**.

**Table 3:** Basic information on AEs related to disulfiram.

Factors	Number of events (%)
Gender	
Female	173 (34.1)
Male	298 (58.7)
Unknown	37 (7.3)
Age	
<17	8 (1.6)
18~64	369 (72.6)
65~85	41 (8.1)
Unknown	90 (17.7)
Reporter	
Consumer	112 (22.0)
Health professionals	98 (19.3)
Lawyer	4 (0.8)
Other health professionals	79 (15.6)
Pharmacist	37 (7.3)
Physician	137 (27.0)
Unknown	41 (8.1)
Reported countries	
Denmark	17 (3.3)
India	34 (6.7)
Italy	18 (3.5)
Sweden	40 (11.6)
Switzerland	19 (3.7)
The United Kingdom	34 (6.7)
The United States	226 (44.5)
Report year	
2004	2 (0.4)
2005	1 (0.2)
2006	4 (0.8)
2007	12 (2.4)
2008	17 (3.3)
2009	16 (3.1)
2010	13 (2.6)
2011	13 (2.6)
2012	9 (1.8)
2013	24 (4.7)
2014	51 (10.0)
2015	60 (11.8)
2016	44 (8.7)
2017	10 (2.0)
2018	29 (5.7)
2019	32 (6.3)
2020	39 (7.7)
2021	79 (15.6)
2022	32 (6.3)
2023	21 (4.1)
Serious outcomes	
Death	25 (3.8)
Disability	24 (3.6)
Hospitalization - initial or prolonged	241 (36.5)
Life-threatening	47 (7.1)
Other serious	255 (38.6)
Missing	52 (7.9)

AE, adverse event.

### 3.2 Disulfiram signal mining

This study identified the intensity of signals and the quantity of reports for disulfiram at the SOC level. Statistically, we identified that disulfiram-induced AEs were associated with 25 SOCs. The SOCs with the highest report frequencies, as shown in **Table 4**, were nervous system disorders(n = 395, ROR 2.64, PRR 2.32, IC 1.21, EBGM 323.48), psychiatric disorders(n = 273, ROR 2.62, PRR 2.40, IC 1.26, EBGM 236.23), general disorders and administration site conditions(n = 268, ROR 0.74, PRR 0.77, IC -0.38, EBGM 22.13), injury, poisoning and procedural complications(n = 176, ROR 0.86, PRR 0.87, IC -0.20, EBGM 3.65) and investigations(n = 168, ROR 1.39, PRR 1.36, IC 0.44, EBGM 16.92), which are consistent with the information recorded in disulfiram’s prescribing information. Notably, the SOC with significant association to disulfiram AEs by meeting all four criteria simultaneously was hepatobiliary disorders (n = 141, ROR 8.26, PRR 7.75, IC 2.95, EBGM 836.51). Besides, this study also revealed unexpected AEs related to gastrointestinal disorders(n = 109, ROR 0.62, PRR 0.64, IC -0.64, EBGM 23.92), metabolism and nutrition disorders(n = 61, ROR 1.42, PRR 1.41, IC 0.49, EBGM 7.33), and vascular and lymphatic vessel diseases(n = 58, ROR 1.36, PRR 1.35, IC 0.43, EBGM 5.39), which were not included in the drug leaflet.

**Table 4:** The signal strength of AEs of disulfiram at the SOC level in the FAERS database.

System organ class	Case Reports	ROR(95% CI)	PRR(χ^2^)	IC(IC025)	EBGM(EBGM05)
Nervous system disorders	395	2.64(2.37-2.95)	2.32(323.48)	1.21(-0.45)	323.48(289.75)
Psychiatric disorders	273	2.62(2.31-2.98)	2.40(236.23)	1.26(-0.40)	236.23(207.91)
General disorders and administration site conditions	268	0.74(0.65-0.84)	0.77(22.13)	-0.38(-2.04)	22.13(19.46)
Injury, poisoning and procedural complications	176	0.86(0.74-1.00)	0.87(3.65)	-0.20(-1.86)	3.65(3.13)
Investigations	168	1.39(1.19-1.63)	1.36(16.92)	0.44(-1.23)	16.92(14.45)
Hepatobiliary disorders	141	8.26(6.96-9.81)	7.75(836.51)	2.95(1.29)	836.51(704.86)
Gastrointestinal disorders	109	0.62(0.51-0.75)	0.64(23.92)	-0.64(-2.31)	23.92(19.72)
Metabolism and nutrition disorders	61	1.42(1.10-1.83)	1.41(7.33)	0.49(-1.17)	7.33(5.68)
Vascular and lymphatic vessel diseases	58	1.36(1.05-1.77)	1.35(5.39)	0.43(-1.23)	5.39(4.15)
Skin and subcutaneous tissue disorders	51	0.46(0.35-0.61)	0.48(31.01)	-1.07(-2.74)	31.01(23.49)
Musculoskeletal and connective tissue disorders	49	0.45(0.34-0.60)	0.47(31.28)	-1.10(-2.76)	31.28(23.56)
Cardiac disorders	43	0.81(0.60-1.09)	0.81(1.95)	-0.30(-1.97)	1.95(1.44)
Respiratory, thoracic and mediastinal disorders	36	0.37(0.27-0.52)	0.38(37.49)	-1.38(-3.05)	37.49(26.97)
Surgical and medical procedures	27	1.03(0.71-1.51)	1.03(0.03)	0.04(-1.62)	0.03(0.02)
Eye disorders	27	0.68(0.47-1.00)	0.69(3.94)	-0.54(-2.21)	3.94(2.70)
Renal and urinary disorders	25	0.65(0.43-0.96)	0.65(4.82)	-0.62(-2.29)	4.82(3.25)
Blood and lymphatic system disorders	25	0.74(0.50-1.10)	0.74(2.25)	-0.43(-2.09)	2.25(1.51)
Infections and infestations	17	0.16(0.10-0.25)	0.16(77.11)	-2.62(-4.28)	77.11(47.84)
Social circumstances	16	1.74(1.06-2.84)	1.73(4.97)	0.79(-0.88)	4.97(3.04)
Immune system disorders	10	0.45(0.24-0.84)	0.46(6.57)	-1.13(-2.80)	6.57(3.53)
Ear and labyrinth disorders	7	0.80(0.38-1.69)	0.80(0.33)	-0.31(-1.98)	0.33(0.16)
Reproductive system and breast disorders	6	0.33(0.15-0.74)	0.33(8.04)	-1.58(-3.25)	8.04(3.61)
Product issues	4	0.13(0.05-0.34)	0.13(24.07)	-2.96(-4.63)	24.07(9.02)
Neoplasms benign, malignant and unspecified (incl cysts and polyps)	2	0.04(0.01-0.15)	0.04(50.91)	-4.74(-6.41)	50.91(12.72)
Pregnancy, puerperium and perinatal conditions	1	0.11(0.02-0.82)	0.12(6.82)	-3.12(-4.78)	6.82(0.96)

AE, adverse event; CI, confidence interval; EBGM, empirical Bayesian geometric mean; EEBGM05, the lower limit of the 95% CI, for EBGM; IC, information component; IC025, the lower limit of 95% CI, for the IC; PRR, proportional reporting ratio; ROR, reporting odds ratio; SOC, system organ class; χ2, chi-squared.

In the comprehensive detection of disulfiram-induced adverse events at the PT level using four different algorithms, a total of 104 AEs were identified. Based on the value of EBGM05 (the most stringent algorithm), the top 50 PTs were listed in **Table 5** (17). In our study, some PTs were consistent with warnings in instructions and drug labeling, such as psychiatric symptom (n = 42, ROR 175.24, PRR 171.57, IC 7.41, EBGM 7076.63), liver transplant (n = 12, ROR 103.55, PRR 102.93, IC 6.68, EBGM 1206.59), acute hepatic failure (n = 17, ROR 40.82, PRR 40.48, IC 5.34, EBGM 653.73), polyneuropathy (n = 13, ROR 35.43, PRR 35.21, IC 5.14, EBGM 431.58), hepatitis acute (n = 8, ROR 38.25, PRR 38.10, IC 5.25, EBGM 288.60) and generalized tonic-clonic seizure (n = 17, ROR 20.12, PRR 19.96, IC 4.32, EBGM 306.02). Notably, off label use (n = 80, ROR 3.31, PRR 3.22, IC 1.69, EBGM 123.94) ranked first occurrence. In addition, a number of AEs were observed that showed significant signal strength, although at low frequencies, including electrocardiogram st segment depression (n = 4, ROR 52.36, PRR 52.26, IC 5.70, EBGM 200.73), delirium (n = 15, ROR 13.90, PRR 13.80, IC 3.79, EBGM 178.07), vocal cord paralysis (n = 3, ROR 50.87, PRR 50.80, IC 5.66, EBGM 146.17), hypomania (n = 4, ROR 32.63, PRR 32.57, IC 5.02, EBGM 122.25), Jarisch-herxheimer reaction (n = 3, ROR 131.57, PRR 131.38, IC 7.03, EBGM 386.22) and so on. These AEs were not documented in the prescribing information and might represent new potential AE signals.

**Table 5:** The top 50 signal strength of AEs of disulfiram at the PTs level in the FAERS database.

SOC	PTs	CaseReports	ROR(95% CI)	PRR(χ2)	IC(IC025)	EBGM(EBGM05)
Metabolism and nutrition disorders	Alcohol intolerance	19	861.70(544.49-1363.71)	853.50(15667.22)	9.69(8.02)	15667.22(10670.26)
Psychiatric disorders	Psychiatric symptom	42	175.24(128.96-238.13)	171.57(7076.63)	7.41(5.75)	7076.63(5475.12)
General disorders and administration site conditions	Alcohol interaction	19	289.55(183.84-456.04)	286.80(5352.72)	8.15(6.48)	5352.72(3660.17)
Nervous system disorders	Wernicke's encephalopathy	6	355.16(158.51-795.79)	354.10(2084.38)	8.45(6.78)	2084.38(1061.22)
Surgical and medical procedures	Liver transplant	12	103.55(58.64-182.84)	102.93(1206.59)	6.68(5.01)	1206.59(749.77)
Nervous system disorders	Toxic encephalopathy	11	73.15(40.41-132.41)	72.75(776.31)	6.18(4.51)	776.31(472.49)
Hepatobiliary disorders	Acute hepatic failure	17	40.82(25.32-65.82)	40.48(653.73)	5.34(3.67)	653.73(438.31)
Hepatobiliary disorders	Jaundice	24	26.48(17.70-39.61)	26.17(580.69)	4.71(3.04)	580.69(414.57)
Nervous system disorders	Polyneuropathy	13	35.43(20.53-61.15)	35.21(431.58)	5.14(3.47)	431.58(273.37)
Immune system disorders	Jarisch-herxheimer reaction	3	131.57(42.28-409.47)	131.38(386.22)	7.03(5.36)	386.22(149.37)
Investigations	Liver function test increased	15	24.79(14.91-41.20)	24.61(339.50)	4.62(2.95)	339.50(221.90)
Nervous system disorders	Peripheral motor neuropathy	4	87.25(32.66-233.07)	87.07(339.22)	6.44(4.77)	339.22(149.08)
Nervous system disorders	Axonal neuropathy	3	111.58(35.87-347.08)	111.41(326.87)	6.79(5.12)	326.87(126.47)
Investigations	Blood alcohol increased	3	104.59(33.63-325.31)	104.44(306.12)	6.70(5.03)	306.12(118.45)
Nervous system disorders	Generalised tonic-clonic seizure	17	20.12(12.48-32.44)	19.96(306.02)	4.32(2.65)	306.02(205.21)
Hepatobiliary disorders	Hepatitis acute	8	38.25(19.09-76.62)	38.10(288.60)	5.25(3.58)	288.60(161.36)
Psychiatric disorders	Psychotic disorder	17	17.61(10.92-28.38)	17.46(263.81)	4.13(2.46)	263.81(176.91)
Nervous system disorders	Encephalopathy	15	19.33(11.63-32.13)	19.19(258.59)	4.26(2.59)	258.59(169.03)
Nervous system disorders	Peripheral sensorimotor neuropathy	3	87.96(28.29-273.48)	87.83(256.67)	6.45(4.78)	256.67(99.35)
Nervous system disorders	Peripheral sensory neuropathy	7	38.78(18.45-81.50)	38.65(256.38)	5.27(3.60)	256.38(137.72)
Hepatobiliary disorders	Ocular icterus	6	41.66(18.68-92.91)	41.54(237.04)	5.37(3.71)	237.04(121.17)
Nervous system disorders	Neuropathy peripheral	29	9.88(6.85-14.25)	9.75(227.95)	3.28(1.62)	227.95(167.72)
Psychiatric disorders	Catatonia	6	37.39(16.77-83.37)	37.28(211.54)	5.22(3.55)	211.54(108.14)
Investigations	Electrocardiogram st segment depression	4	52.36(19.61-139.80)	52.26(200.73)	5.70(4.04)	200.73(88.26)
General disorders and administration site conditions	Drug interaction	37	7.22(5.22-10.00)	7.11(194.65)	2.83(1.16)	194.65(148.27)
Hepatobiliary disorders	Hepatic necrosis	5	38.22(15.88-91.97)	38.12(180.49)	5.25(3.58)	180.49(86.56)
Psychiatric disorders	Delirium	15	13.90(8.36-23.10)	13.80(178.07)	3.79(2.12)	178.07(116.40)
Surgical and medical procedures	Self-medication	5	37.69(15.66-90.70)	37.60(177.87)	5.23(3.56)	177.87(85.30)
Hepatobiliary disorders	Drug-induced liver injury	13	15.68(9.09-27.06)	15.59(177.43)	3.96(2.29)	177.43(112.41)
Nervous system disorders	Neurotoxicity	10	19.40(10.42-36.12)	19.31(173.51)	4.27(2.60)	173.51(103.14)
Hepatobiliary disorders	Hepatitis toxic	4	45.20(16.93-120.66)	45.11(172.26)	5.49(3.82)	172.26(75.75)
Investigations	Hepatic enzyme increased	21	10.04(6.53-15.44)	9.95(169.15)	3.31(1.65)	169.15(118.03)
Hepatobiliary disorders	Hepatic failure	14	14.04(8.30-23.76)	13.95(168.34)	3.80(2.13)	168.34(108.42)
General disorders and administration site conditions	Food interaction	3	51.47(16.57-159.91)	51.40(147.96)	5.68(4.01)	147.96(57.31)
Nervous system disorders	Vocal cord paralysis	3	50.87(16.38-158.05)	50.80(146.17)	5.66(4.00)	146.17(56.62)
Psychiatric disorders	Delusion	9	18.16(9.43-34.97)	18.08(145.19)	4.18(2.51)	145.19(83.93)
Investigations	Electroencephalogram abnormal	4	37.22(13.95-99.34)	37.15(140.51)	5.21(3.55)	140.51(61.80)
Hepatobiliary disorders	Liver injury	10	15.71(8.44-29.25)	15.64(136.97)	3.97(2.30)	136.97(81.42)
Psychiatric disorders	Mental status changes	12	13.10(7.43-23.11)	13.03(133.27)	3.70(2.04)	133.27(82.88)
Blood and lymphatic system disorders	Methaemoglobinaemia	3	44.03(14.17-136.76)	43.96(125.75)	5.46(3.79)	125.75(48.71)
Injury, poisoning and procedural complications	Off label use	80	3.31(2.65-4.14)	3.22(123.94)	1.69(0.02)	123.94(102.79)
Injury, poisoning and procedural complications	Wrong dose	3	43.04(13.86-133.69)	42.98(122.80)	5.42(3.75)	122.80(47.57)
Psychiatric disorders	Hypomania	4	32.63(12.23-87.08)	32.57(122.25)	5.02(3.36)	122.25(53.77)
Injury, poisoning and procedural complications	Toxicity to various agents	32	5.52(3.90-7.83)	5.45(116.64)	2.45(0.78)	116.64(87.08)
Social circumstances	Alcohol use	4	29.92(11.21-79.84)	29.86(111.46)	4.90(3.23)	111.46(49.03)
Investigations	Alanine aminotransferase increased	17	8.31(5.16-13.40)	8.25(108.35)	3.04(1.38)	108.35(72.66)
Nervous system disorders	Posterior reversible encephalopathy syndrome	6	18.96(8.51-42.27)	18.91(101.71)	4.24(2.57)	101.71(52.01)
Hepatobiliary disorders	Hepatic function abnormal	12	10.41(5.90-18.36)	10.35(101.41)	3.37(1.70)	101.41(63.07)
Psychiatric disorders	Alcoholism	4	26.22(9.83-69.97)	26.17(96.74)	4.71(3.04)	96.74(42.56)
Investigations	Transaminases increased	9	12.40(6.44-23.86)	12.34(93.82)	3.63(1.96)	93.82(54.24)

AE, adverse event; CI, confidence interval; EBGM, empirical Bayesian geometric mean; EEBGM05, the lower limit of the 95% CI, for EBGM; IC, information component; IC025, the lower limit of 95% CI, for the IC; PRR, proportional reporting ratio; PT: preferred term; ROR, reporting odds ratio; SOC, system organ class; χ2, chi-squared.

## 4 Discussion

Disulfiram, the first FDA-approved drug to treat AUD, has been used since 1951. Due to the disulfiram-ethanol reaction, alcohol and a variety of household products must be avoided during disulfiram treatment. Additionally, disulfiram can cause multiple side effects and affect the metabolism of other drugs by inhibiting cytochrome P450 reductase. Disulfiram’s table information lists common side effects such as headaches, drowsiness, fatigue, and a metallic or garlic-like taste (18). Both the insert and randomized controlled trials report more severe reactions like optic neuritis, peripheral neuropathy, and hepatotoxicity, which are more frequent at higher doses or when combined with other medications (19, 20). These factors contribute to poor patient compliance, leading to a gradual decline in the use of disulfiram. However, in recent years, disulfiram has shown new progress in novel therapeutic areas such as antitumor therapy (10). This study identified a series of disulfiram-related AEs signals and systematically evaluated its real-world safety, providing a reference for its application in new indications.

### 4.1 Basic analysis of AE occurrences

This study examined disulfiram AE reports from the FAERS database over the last two decades. The higher proportion of male reporters could be linked to a higher prevalence of AUD among men. According to research, the frequency of AUD among males is about 4-5 times higher than in females (21). From the perspective of reported age, it partly reflects that the primary population for AUD consists of adults (21), and partly relates to the broad age range covered, suggesting that further stratification by age may be required in future analyses. The gradual decline in AE reports over the past five years might be influenced by the reduced use of disulfiram in the treatment of AUD (3). Physicians constitute the largest reporting group, possibly indicating close attention from healthcare professionals to drug reactions. Given that most reports come from the U.S. (44.5%), this is likely related to the country’s relatively high prevalence of AUD. According to the 2023 National Survey on Drug Use and Health (NSDUH), approximately 10.2% of the U.S. population had AUD, ranking the country 5th among those with the highest AUD prevalence (22, 23). Over the past decade, the number of disulfiram adverse event reports has fluctuated, influenced by the decline in its use for traditional indications and the exploration of new indications, particularly with a notable increase in 2021 due to trials in anti-tumor therapy (9). The high incidence of hospitalization or prolonged hospital stays may imply negative impacts of disulfiram on patient outcomes, underscoring the need for heightened vigilance in monitoring adverse reactions during clinical use.

### 4.2 Known AEs

#### 4.2.1 Hepatobiliary AEs

Due to the biological and histological changes in the liver caused by unhealthy alcohol use in most patients who used disulfiram, the true incidence of disulfiram-induced hepatotoxicity was unclear. Based on data from the Swedish DILI (Drug-Induced Liver Injury) registry, Björnsson et al. identified 82 reports of disulfiram-induced liver injury over 36 years. Specifically, approximately 1 case of liver injury was reported per 1.3 million average daily doses of disulfiram (24). The risk of severe hepatitis leading to death or requiring liver transplantation was even lower. According to Chick’s estimates, only 1 fatal case occurs annually per 30,000 patients treated with disulfiram (25). The mechanism of disulfiram-induced liver injury was not fully understood, but it was primarily associated with its metabolites inhibiting the P4502E1 enzyme and the production of autoantibodies against cytochrome P450 enzymes. The autoimmune response resulted in a hypersensitivity reaction characterized by eosinophilic infiltration in the liver. Additionally, a few liver biopsy specimens showed hepatocellular drop-out necrosis, which was associated with worse outcomes such as liver transplantation or fulminant liver failure. A study on biomarkers for drug-induced liver injury indicated that bilirubin levels could predict death or liver transplantation (26). This study suggested that clinicians should regularly monitor liver function when administering disulfiram.

#### 4.2.2 Neuropathy AEs

Due to the potential risk of unexpected disulfiram-alcohol reaction, disulfiram drug labeling recommendations require extra caution when prescribing disulfiram for patients with conditions such as epilepsy. In reality, disulfiram could cause seizures even without alcohol consumption. Disulfiram inhibits dopamine-β-hydroxylase, an enzyme that converts dopamine to norepinephrine. The inhibition results in an imbalance of neurotransmitters, namely an increase in dopamine levels and a decrease in norepinephrine levels in the brain. This imbalance directly affects the threshold for seizures (27, 28). In addition, studies have demonstrated that carbon disulfide (CS2), which is another byproduct of disulfiram metabolism, can cause seizures in both animals and people (29). Disulfiram may have reduced the seizure threshold, resulting in more frequent and severe seizures, particularly generalized tonic-clonic seizures (30). The prescribing instructions of disulfiram also documented peripheral neuropathy as an adverse effect, so indirectly corroborating the credibility of this study.

### 4.3 Potential mechanisms of new potential AEs

#### 4.3.1 Neurological and Psychiatric AEs

Hypomania was related to the increase in dopamine concentration in the brain caused by disulfiram (31). The heightened activity of dopamine within the mesolimbic system, particularly in the nucleus accumbens and ventral tegmental area, could potentially explain the emergence of hypomanic moods. Delirium was one of the clinical manifestations of disulfiram encephalopathy, although the exact mechanism remained unclear. The toxic metabolite of disulfiram, diethyldithiocarbamate, precipitated upon binding with copper. The excess dopamine, copper deposition in the basal ganglia, and CS2 accumulation might have contributed to encephalopathy development. Vocal cord paralysis was also identified as a neurological complication induced by disulfiram. The potential mechanisms for this condition include the following: On one hand, CS2, a known axonal toxin, could cause neuropathological changes (29). On the other hand, disulfiram might directly affect Schwann cells, disrupting the formation of the myelin sheath around nerve fibers (32, 33). Although neurological adverse effects were rare, they had the potential to cause severe consequences once they occurred. In clinical practice, the identification and management of these neurological adverse effects required a clear understanding of the clinical manifestations and timely implementation of appropriate interventions.

#### 4.3.2 Cardiac system AEs

Electrocardiogram st segment depression was associated with the disulfiram-alcohol reaction. The disulfiram-alcohol reaction referred to a series of adverse symptoms resulting from the accumulation of acetaldehyde in the body due to disulfiram’s inhibition of ALDH activity. Typical symptoms included flushing, headache, nausea, vomiting, sweating, dizziness, and vertigo. More severe cases might present with profound hypotension, arrhythmias, myocardial infarction, and cardiovascular collapse (34). Currently, disulfiram was forbidden in patients with severe myocardial disease or coronary occlusion. It emphasized the importance of ensuring that patients avoid ethanol and other interacting substances while on disulfiram treatment.

#### 4.3.3 Off label use-related AEs

Disulfiram was originally indicated for the treatment of AUD to aid in abstinence. Recently, growing evidence has demonstrated the potential of repurposing disulfiram for the treatment of various pathologies such as inflammation, Lyme disease, and cancer (4, 10, 35). Numerous mechanistic studies have shown that disulfiram possesses remarkable anticancer properties, such as triggering oxidative stress (36), inhibiting proteasome activity (37), reducing angiogenesis (38), blocking the cell cycle (36), decreasing cancer stemness (39), reversing drug resistance (40), limiting tumor metastasis (41), and modulating the immune microenvironment (42). Reports of off label use of disulfiram have been substantial. However, the drug’s clinical utility has been hampered by its extensive adverse effects. A case involving off-label disulfiram use for melanoma described the onset of posterior reversible encephalopathy syndrome (PERS), a rare but severe side effect, after two weeks of treatment (43). The Jarisch-Herxheimer reaction has also been associated with off-label use of disulfiram in treating Lyme disease (4). This finding highlights the significant risks associated with off-label use, which currently lacks sufficient clinical research support.

### 4.4 Limitations

This study conducted a detailed analysis of the FAERS database to explore AEs closely associated with disulfiram use. However, the study’s limitations must be acknowledged. The FAERS database observational reports, upon which this investigation was based, do not prove a direct correlation between the medicine and the side effects. To identify the exact processes causing these events, more clinical trials and preclinical investigations are required. It is possible that preexisting diseases, rather than the medicine itself, were to blame for the reported side effects. Few reports of AEs were included in the study because it relied on participants’ own words to fill out the questionnaires. Compared to randomized controlled trials, the FAERS data has inherent limitations such as reporting bias and underreporting. However, it provides valuable real-world insights into adverse events that may not be fully captured in the controlled environments of controlled trials. In addition, the drug’s characteristics, individual patient characteristics, and other medical issues can all impact the signal for disulfiram-related adverse reactions. These confounding factors have the potential to affect the reliability of the study’s results. A thorough assessment of disulfiram’s safety should be conducted in future studies by combining extensive clinical evaluations with long-term data.

## 5 Conclusion

This research discovered some anticipated and unforeseen adverse effects of disulfiram through their examination of the FAERS database. The reported AEs, including neurotoxicity and hepatotoxicity, are consistent with the information recorded in the disulfiram prescribing information, validating the reliability of this study. The frequent utilization of disulfiram for off-label purposes highlights the drug’s capacity for repurposing. Delirium, vocal cord paralysis, and electrocardiogram st segment depression were among the many possible AEs found during the investigation that were not mentioned on the drug’s label. Although there are some limitations to the data, these preliminary findings will certainly be valuable for monitoring the safety of disulfiram in clinical practice. The study also points the way towards potential areas of investigation for the future. Exploring the particular mechanisms and management techniques for disulfiram-related AEs is vital for developing disulfiram’s new clinical applications.

## Data Availability

The original contributions presented in the study are included in the article/supplementary material. Further inquiries can be directed to the corresponding author.

## References

[1] Kranzler HR. Overview of alcohol use disorder. Am J Psychiatry. (2023) 180:565–72. doi: 10.1176/appi.ajp.20230488 37525595

[2] Lanz J Biniaz-Harris N Kuvaldina M Jain S Lewis K Fallon BA. Disulfiram: mechanisms, applications, and challenges. Antibiotics. (2023) 12:524. doi: 10.3390/antibiotics12030524 36978391 PMC10044060

[3] Ehrie J Hartwell EE Morris PE Mark TL Kranzler HR. Survey of addiction specialists’ use of medications to treat alcohol use disorder. Front Psychiatry. (2020) 11:47. doi: 10.3389/fpsyt.2020.00047 32116860 PMC7034336

[4] Gao J Gong Z Montesano D Glazer E Liegner K. Repurposing” disulfiram in the treatment of lyme disease and babesiosis: retrospective review of first 3 years’ experience in one medical practice. Antibiotics. (2020) 9:868. doi: 10.3390/antibiotics9120868 33291557 PMC7761882

[5] Huang L Zhu J Wu G Xiong W Feng J Yan C. A strategy of “adding fuel to the flames” enables a self-accelerating cycle of ferroptosis-cuproptosis for potent antitumor therapy. Biomaterials. (2024) 311:122701. doi: 10.1016/j.biomaterials.2024.122701 38981152

[6] Xu J Pickard JM Núñez G. FDA-approved disulfiram inhibits the NLRP3 inflammasome by regulating NLRP3 palmitoylation. Cell Rep. (2024) 43:114609. doi: 10.1016/j.celrep.2024.114609 39116210 PMC11398858

[7] Mego M Svetlovska D Angelis V D Kalavska K Lesko P Makovník M. Phase II study of disulfiram and cisplatin in refractory germ cell tumors. The GCT-SK-006 phase II trial. Investigational New Drugs. (2022) 40:1080–6. doi: 10.1007/s10637-022-01271-1 35763178

[8] Werlenius K Kinhult S Solheim TS Magelssen H Löfgren D Mudaisi M. Effect of disulfiram and copper plus chemotherapy vs chemotherapy alone on survival in patients with recurrent glioblastoma: A randomized clinical trial. JAMA Network Open. (2023) 6:e234149. doi: 10.1001/jamanetworkopen.2023.4149 37000452 PMC10066460

[9] Huang J Campian JL DeWees TA Skrott Z Mistrik M Johanns TM. A phase 1/2 study of disulfiram and copper with concurrent radiation therapy and temozolomide for patients with newly diagnosed glioblastoma. Int J Radiat Oncology Biology Phys. (2024) 120:738–49. doi: 10.1016/j.ijrobp.2024.05.009 PMC13123272 38768767

[10] Zhou Z Zhou C Liu J Yuan Y Yao C Liu M. Tumor specific in *situ* synthesis of therapeutic agent for precision cancer therapy. J Nanobiotechnology. (2024) 22:612. doi: 10.1186/s12951-024-02825-6 39385273 PMC11465910

[11] Ghosh A Mahintamani T Balhara YPS Roub FE Basu D Bn S. Disulfiram ethanol reaction with alcohol-based hand sanitizer: an exploratory study. Alcohol Alcoholism. (2021) 56:42–6. doi: 10.1093/alcalc/agaa096 PMC7665335 33150930

[12] Greil W Zhang X Stassen H Grohmann R Bridler R Hasler G. Cutaneous adverse drug reactions to psychotropic drugs and their risk factors—A case-control study. Eur Neuropsychopharmacol. (2019) 29:111–21. doi: 10.1016/j.euroneuro.2018.10.010 30424913

[13] Brown EG. Using MedDRA: Implications for risk management. Drug Saf. (2004) 27:591–602. doi: 10.2165/00002018-200427080-00010 15154830

[14] Bate A Lindquist M Edwards IR Olsson S Orre R Lansner A. A Bayesian neural network method for adverse drug reaction signal generation. Eur J Clin Pharmacol. (1998) 54:315–21. doi: 10.1007/s002280050466 9696956

[15] Evans SJ Waller PC Davis S. Use of proportional reporting ratios (PRRs) for signal generation from spontaneous adverse drug reaction reports. Pharmacoepidemiology Drug Saf. (2001) 10:483–6. doi: 10.1002/pds.677 11828828

[16] Rothman KJ Lanes S Sacks ST. The reporting odds ratio and its advantages over the proportional reporting ratio. Pharmacoepidemiology Drug Saf. (2004) 13:519–23. doi: 10.1002/pds.1001 15317031

[17] Sakaeda T Tamon A Kadoyama K Okuno Y. Data mining of the public version of the FDA adverse event reporting system. Int J Med Sci. (2013) 10:796–803. doi: 10.7150/ijms.6048 23794943 PMC3689877

[18] DailyMed — DISULFIRAM tablet. Available online at: https://dailymed.nlm.nih.gov/dailymed/drugInfo.cfm?setid=b90f8ff4-4fb3-46ab-8149-cb55f24a4044 (accessed October 21, 2024).

[19] Malcolm R Olive MF Lechner W. The safety of disulfiram for the treatment of alcohol and cocaine dependence in randomized clinical trials: Guidance for clinical practice. Expert Opin Drug Saf. (2008) 7:459–72. doi: 10.1517/14740338.7.4.459 18613809

[20] Sinclair JMA Chambers SE Shiles CJ Baldwin DS. Safety and tolerability of pharmacological treatment of alcohol dependence: Comprehensive review of evidence. Drug Saf. (2016) 39:627–45. doi: 10.1007/s40264-016-0416-y 27023898

[21] MacKillop J Agabio R Feldstein-Ewing S Heilig M Kelly JF Leggio L. Hazardous drinking and alcohol use disorders. Nat Rev. (2022) 8:80. doi: 10.1038/s41572-022-00406-1 PMC10284465 36550121

[22] Alcoholism by country 2024 . Available online at: https://worldpopulationreview.com/country-rankings/alcoholism-by-country (accessed October 19, 2024).

[23] Understanding alcohol use disorder | national institute on alcohol abuse and alcoholism (NIAAA). Available online at: https://www.niaaa.nih.gov/publications/brochures-and-fact-sheets/understanding-alcohol-use-disorder (accessed October 19, 2024).

[24] Björnsson E Nordlinder H Olsson R. Clinical characteristics and prognostic markers in disulfiram-induced liver injury. J Hepatol. (2006) 44:791–7. doi: 10.1016/j.jhep.2005.12.016 16487618

[25] Chick J. Safety issues concerning the use of disulfiram in treating alcohol dependence. Drug Saf. (1999) 20:427–35. doi: 10.2165/00002018-199920050-00003 10348093

[26] Chiang H-C Wu I-C. Useful biomarkers for predicting poor prognosis of patients with drug-induced liver injury: A retrospective cohort study. Am J Med Sci. (2024) S0002-9629:01412–5. doi: 10.1016/j.amjms.2024.08.019 39182648

[27] Akyuz E Polat AK Eroglu E Kullu I Angelopoulou E Paudel YN. Revisiting the role of neurotransmitters in epilepsy: An updated review. Life Sci. (2021) 265:118826. doi: 10.1016/j.lfs.2020.p.118826 33259863

[28] Frankowska M Surówka P Suder A Pieniążek R Pukło R Jastrzębska J. Treatment with dopamine β-hydroxylase (DBH) inhibitors prevents morphine use and relapse-like behavior in rats. Pharmacol Rep. (2021) 73:1694–711. doi: 10.1007/s43440-021-00307-2 PMC8599263 34236605

[29] Printemps N Le Magueresse-Battistoni B Mhaouty-Kodja S Viguié C Michel C. How to differentiate general toxicity-related endocrine effects from endocrine disruption: systematic review of carbon disulfide data. Int J Mol Sci. (2022) 23:3153. doi: 10.3390/ijms23063153 35328575 PMC8952789

[30] Vaidyanathan S Raghunathan S Udupa ST Munoli RN Manjushree MS Praharaj SK. Disulfiram-associated generalized tonic-clonic seizures. Am J Ther. (2024) 31:e422–6. doi: 10.1097/MJT.0000000000001625 38976526

[31] González-Romero MF Martínez-Ávila JM Moloney E García-Cabeza I. Aversive agents: Think twice. A case report on disulfiram-induced mania. Bipolar Disord. (2024) 26:395–7. doi: 10.1111/bdi.13397 38015080

[32] De Logu F Li Puma S Landini L Portelli F Innocenti A de Araujo DSM. Schwann cells expressing nociceptive channel TRPA1 orchestrate ethanol-evoked neuropathic pain in mice. J Clin Invest. (2019) 129:5424–41. doi: 10.1172/JCI128022 PMC6877331 31487269

[33] Utrera J Romero R Verdaguer E Junyent F Auladell C. Recovery of axonal myelination sheath and axonal caliber in the mouse corpus callosum following damage induced by N, N-diethyldithiocarbamate. Eur J Neurosci. (2011) 34:2007–14. doi: 10.1111/j.1460-9568.2011.07928.x 22132728

[34] Amuchastegui T Amuchastegui M Donohue T. Disulfiram—Alcohol reaction mimicking an acute coronary syndrome. Connecticut Med. (2014) 78:81–4. 24741856

[35] Guo W Chen S Li C Xu J Wang L. Application of disulfiram and its metabolites in treatment of inflammatory disorders. Front Pharmacol. (2021) 12:795078. doi: 10.3389/fphar.2021.795078 35185542 PMC8848744

[36] Hassani S Ghaffari P Chahardouli B Alimoghaddam K Ghavamzadeh A Alizadeh S. Disulfiram/copper causes ROS levels alteration, cell cycle inhibition, and apoptosis in acute myeloid leukaemia cell lines with modulation in the expression of related genes. Biomedicine Pharmacotherapy. (2018) 99:561–9. doi: 10.1016/j.biopha.2018.01.109 29902866

[37] Chen C Nie D Huang Y Yu X Chen Z Zhong M. Anticancer effects of disulfiram in T-cell Malignancies through NPL4-mediated ubiquitin-proteasome pathway. J Leukocyte Biol. (2022) 112:919–29. doi: 10.1002/JLB.5MA1121-644R 35363385

[38] Roy B Palaniyandi SS. Aldehyde dehydrogenase 2 inhibition potentiates 4-hydroxy-2-nonenal induced decrease in angiogenesis of coronary endothelial cells. Cell Biochem Funct. (2020) 38:290–9. doi: 10.1002/cbf.3468 31943249

[39] Guo F Yang Z Sehouli J Kaufmann AM. Blockade of ALDH in cisplatin-resistant ovarian cancer stem cells in *vitro* synergistically enhances chemotherapy-induced cell death. Curr Oncol. (2022) 29:2808–22. doi: 10.3390/curroncol29040229 PMC9031660 35448203

[40] Kita Y Hamada A Saito R Teramoto Y Tanaka R Takano K. Systematic chemical screening identifies disulfiram as a repurposed drug that enhances sensitivity to cisplatin in bladder cancer: A summary of preclinical studies. Br J Cancer. (2019) 121:1027–38. doi: 10.1038/s41416-019-0609-0 PMC6964684 31673101

[41] Bu W Wang Z Meng L Li X Liu X Chen Y. Disulfiram inhibits epithelial-mesenchymal transition through TGFβ-ERK-Snail pathway independently of Smad4 to decrease oral squamous cell carcinoma metastasis. Cancer Manage Res. (2019) 11:3887–98. doi: 10.2147/CMAR.S199912 PMC6504671 31118804

[42] Hu JJ Liu X Xia S Zhang Z Zhang Y Zhao J. FDA-approved disulfiram inhibits pyroptosis by blocking gasdermin D pore formation. Nat Immunol. (2020) 21:736–45. doi: 10.1038/s41590-020-0669-6 PMC7316630 32367036

[43] Beam DR Knight JM. Posterior reversible encephalopathy syndrome instigated by off-label disulfiram use for metastatic melanoma. Psychosomatics. (2020) 61:302–6. doi: 10.1016/j.psym.2019.09.003 PMC7075719 31668552

